# Research progress of microglial surface receptors in perioperative neurocognitive disorders

**DOI:** 10.1002/ibra.12136

**Published:** 2023-11-30

**Authors:** Chun‐Chun Tang, De‐Xing Liu, Zhao‐Qiong Zhu

**Affiliations:** ^1^ Department of Anesthesiology Affiliated Hospital of Zunyi Medical University Zunyi Guizhou China

**Keywords:** cell surface receptors, microglia, neuroinflammatory, perioperative neurocognitive disorders

## Abstract

Perioperative neurocognitive disorder (PND) is a common complication in the perioperative period, which not only prolongs the hospitalization of patients, increases the cost of treatment, but even increases the postoperative mortality of patients, bringing a heavy burden to families and society. Mechanism exploration involves anesthesia and surgery that lead to microglial activation, promote the synthesis and secretion of inflammatory factors, cause an inflammatory cascade, aggravate nerve cell damage, and lead to cognitive dysfunction. It is believed that microglia‐mediated neuroinflammatory responses play a vital role in the formation of PND. Microglia surface receptors are essential mediators for microglia to receive external stimuli, regulate microglial functional status, and carry out intercellular signal transmission. Various microglial surface receptors trigger neuroinflammation, damage neurons, and participate in the development and progression of PND by activating microglia. In this study, the roles of immunoglobulin receptors, chemokine receptors, purinergic receptors, and pattern recognition receptors in microglia surface receptors in PND were reviewed, to provide a reference for the mechanism research, prevention, and treatment of PND.

## Graphical abstract

Perioperative factors such as surgical trauma, anesthetic, and inflammatory factors act on microglia in the brain. The receptor on microglia is activated to promote or inhibit the occurrence of inflammatory reactions, which, in turn, induce or prevent the occurrence of perioperative neurocognitive disorder (PND).

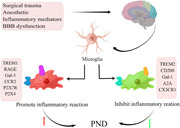

## 1 INTRODUCTION

Perioperative neurocognitive disorder (PND) is one of the most common complications in perioperative patients and includes cognitive function changes before the operation, postoperative delirium, delayed recovery of neurocognitive function, and postoperative cognitive dysfunction.^1^ PND not only prolongs the hospital stay and increases the cost of treatment but also increases the occurrence of other related complications and postoperative mortality of patients;^2^, ^3^ it weighs heavily on family and society. Currently, the pathogenesis of PND is not clear. It is believed that mitochondrial damage, oxidative stress, neuritis, Tau protein phosphorylation, and central cholinergic system disorder are all involved in the damage of brain nerves caused by general anesthesia to varying degrees. Among them, the role of neuroinflammation theory in the pathogenesis of PND has attracted much attention from scholars in recent years.^4^, ^5^, ^6^ Studies have shown that microglia‐mediated neuroinflammatory reaction plays a key role in the formation of PND.^7^, ^8^ Microglial surface receptor is an important medium for microglia to receive external stimuli, regulate the functional state of microglia, and transmit signals between cells. A variety of microglia surface receptors trigger neuroinflammation and damage neurons by activating microglia and participating in the occurrence and development of PND. This study reviews the role of microglia surface receptors in PND, to provide reference for the mechanism study of PND and the discovery of potential biomarkers and new therapeutic targets.

### 1.1 PND

PND is one of the most common complications in perioperative surgical patients, mainly including preoperative cognitive changes, postoperative delirium, delayed recovery of neurocognitive function, and postoperative cognitive dysfunction. The phenomenon of cognitive decline after anesthesia operation has been widely concerned as early as the 1990s. In 1998, Moller and others formally named this phenomenon as postoperative cognitive dysfunction (POCD) in an international multi‐center study. However, the nomenclature has the following drawbacks: (1) the patient's preoperative cognitive impairment was not evaluated; (2) the patient's quality of daily life was not evaluated; and (3) its diagnosis is disconnected from the existing clinical diagnostic standards. Until 2018, experts in various neurocognitive fields, such as anesthesiology, neurology, geriatrics, and psychiatry officially changed the name of POCD to PND after discussion.^1^ The nomenclature includes the concept of POCD and postoperative delirium, which is mainly manifested as dysfunction in multiple cognitive dimensions, such as perioperative attention, mental state, and consciousness level. The latter is expressed in confusion with time and place, character disorientation, difficulty in concentrating, and so on. The incidence of PND varies from 10% to 65% depending on multiple factors^9^ (age, education, sex, and type of surgery) with advanced age being an independent risk factor for PND.^10^ With the advent of an aging society and the rapid development of medical technology, more and more elderly patients have the opportunity to get operated on. As a common perioperative complication, PND not only prolongs the hospitalization time of patients and increases the cost of treatment but also increases the occurrence of other related complications and postoperative mortality of patients, weighing heavily on family and society. Therefore, it is of great significance to prevent and reduce the occurrence of PND in patients.

### 1.2 Microglia and PND

**Figure 1:**
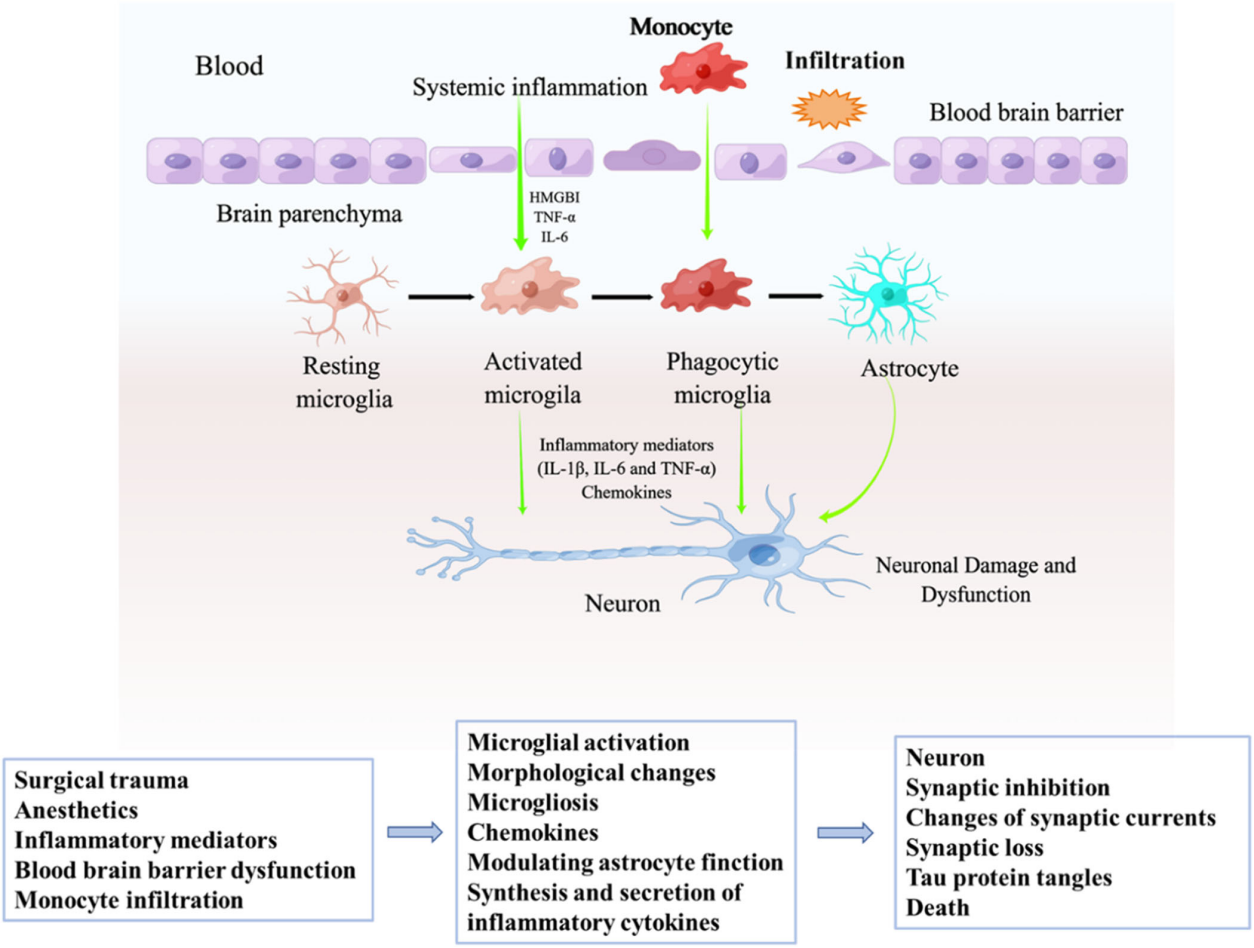
Schematic overview of microglial mechanisms involved in PND. HMGB1, high mobility group box chromosomal protein 1; IL‐1β, interleukin‐1β; IL‐6, interleukin‐ 6; PND, perioperative neurocognitive disorder; TNF‐α, tumor necrosis factor‐α. [Color figure can be viewed at wileyonlinelibrary.com]

Currently, the pathogenesis of PND is unknown. It is believed that mitochondrial damage, oxidative stress, neuroinflammation, Tau protein phosphorylation, central cholinergic system disorders, and so forth are all involved in the damage of brain nerves by general anesthetics to varying degrees, and the role of neuroinflammation theory in the pathogenesis of PND has attracted much attention in recent years. Microglia‐mediated neuroinflammatory responses have been shown to play a crucial role in the formation of PND^11^ (Figure 1).

Microglia are resident immune cells of the brain and significant effector cells in the inflammatory response of the central nervous system.^12^ Microglia are the first and most important line of immune defense^13^ and play a key role in maintaining neuronal homeostasis and synaptic plasticity. It is well known that microglia play a dual role in nerve damage and recovery, performing functions that improve or destroy tissue integrity depending on the cellular environment in which they are located at the time. At rest, microglia are branched and small, monitoring changes in the local microenvironment and detecting damage to the central nervous system. When stimulated or damaged by the outside world, the microglia cell body increases, and the branches become shorter and thicker, showing a typical “amoeba‐like.” So as to be activated into different phenotypes. Among them, the classically activated type (M1 type) is related to the release of a variety of proinflammatory factors, such as inducible nitric oxide synthase (iNOS), tumor necrosis factor‐α (TNF‐α), interleukin‐1β (IL‐1β), and so forth, which promotes inflammation and tissue damage, and produces toxic effects on neurons. Surrogate‐activated (M2 type) is associated with releasing a variety of anti‐inflammatory factors, such as arginase (ARG), transforming growth factor beta (TGF‐β), IL‐10, and so forth, which inhibits inflammation, promotes tissue repair and neuronal regeneration, and avoids secondary inflammatory damage.^14^ Studies have shown that anesthesia and surgery lead to microglial activation, and promote the synthesis and secretion of inflammatory factors (IL‐1β, IL‐6, TNF‐α, etc.) resulting in an inflammatory cascade.^15^

Elderly mice showed significant deficits in learning and memory after anesthesia surgery. At the same time, microglia get activated, and hippocampal TNF‐α and IL‐1β expression increase.^16^ Early microglia depletion reduces anesthesia surgery‐induced secretion of hippocampal inflammatory factors and alleviates cognitive decline in mice.^17^ Plasma levels of proinflammatory cytokines (e.g., TNF‐α, IL‐6) were found to be significantly elevated after surgery, which was associated with the development of PND.^18^ An increase in inflammatory cytokines such as TNF‐α in plasma affects the permeability of the blood‐brain barrier through the NF‐κB pathway, invading the central nervous system and causing neuroinflammation.^19^ The increase in proinflammatory cytokines such as TNF‐α in the brain can activate microglia, which release a variety of proinflammatory cytokines after activation. This positive feedback amplification mechanism of inflammation further aggravates nerve cell damage, leading to cognitive dysfunction.^17^ The above studies suggest that microglia activated in PND mainly secrete pro‐inflammatory cytokines, and M1 microglia dominate in PND. The study found that the M2‐type response of microglia after cerebral ischemia in elderly mice was impaired.^20^ We believe that the important role of M1 microglia in neuroinflammation is one of the factors leading to PND in elderly patients. Amplified neuroinflammation after microglia activation contributes to the development of PND. The above study once again shows that microglia‐mediated neuroinflammatory responses play an irreplaceable role in the formation of PND.

### 1.3 Microglial surface receptors and PND

Microglia surface receptors are important mediators for microglia to receive external stimuli and regulate the functional state of microglia for intercellular signal transmission. It plays an irreplaceable role in PND, Alzheimer's disease (AD), Parkinson's disease, and other neurological diseases. Various microglial surface receptors (immunoglobulin superfamily receptors, chemokine receptors, purinergic receptors, pattern recognition receptors [PRR], etc.) trigger neuroinflammation by activating microglia, damaging neurons, and participating in the initiation and progression of PND (Table 1).^21^ The study of microglial surface receptors is of great significance for understanding the pathogenesis of PND and discovering potential biomarkers and new therapeutic targets.

**Table 1:** Effects of some microglia surface receptor families on PND.

Receptor families	Receptor	Hypotype	Influence on PND	References
IgSF	TREM	TREM1	Promote inflammatory reaction	[22]
		TREM2	Inhibit inflammatory reaction	[23, 24, 25]
	CD200		Maintain the static state of microglia and inhibit inflammatory reaction	[26, 27, 28, 29, 30, 31, 32]
PRR	TLRs	TLR1‐9	This review does not describe	
	RAGE		Promote inflammatory reaction	[33, 34, 35, 36]
	CBR	Gal‐1	Inhibit inflammatory reaction	[37, 38, 39]
		Gal‐3	Promote inflammatory reaction	[37]
Purinergic receptor	P1	A1	Improve synaptic transmission and improve learning and memory ability	[40]
		A2A	Inhibit inflammatory reaction	[41, 42, 43, 44]
	P2	P2X	Promote inflammatory reaction	[45, 46, 47, 48, 49, 50, 51, 52]
Chemokine receptor	CX3CR1		Inhibit inflammatory reaction	[53, 54, 55, 56]
	CCR2		Promote inflammatory reaction	[57, 58]
Cannabinoid receptor			Inhibit inflammatory reaction	[59]
Complement receptor			Unknown	[60]
Histamine receptor			Inhibit inflammatory reaction	[61]

Abbreviations: A1, adenosine receptor A1; A2A, adenosine receptor A2A; CBR, carbohydrate binding receptors; CD200, cluster of differentiation 200 receptor; Gal‐1, galectin1; Gal‐3, galectin3; IgSF, immunoglobulin superfamily receptors; PND, perioperative neurocognitive disorders; PRR, pattern recognition receptor; P2X, Purinergic receptor P2X; RAGE, receptor for advanced glycation endproducts; TLR1‐9, toll‐like receptor 1‐9; TLRs, toll‐like receptors; TREM, triggering receptor expressed on myeloid cells; TREM1, triggering receptor expressed on myeloid cells 1; TREM2, triggering receptor expressed on myeloid cells 1.

## 2 MICROGLIAL RECEPTORS IN THE IMMUNOGLOBULIN SUPERFAMILY

In recent years, the receptors in the immunoglobulin superfamily (IgSF), which consists of proteins with one or more IgSF domains, have attracted much attention in PND. Some members of this family recognize external signals and keep microglia in a normal surveillance state, signaled by the immunoreceptor tyrosine inhibitory motif (ITIM) based on the cytoplasmic domain. Instead, individual receptors in this family sense pathological changes in neurons and other central nervous system components, signaled by the immunoreceptor tyrosine activation motif (ITAM) based on the cytoplasmic domain, leading to microglial activation.^62^ The two types of receptors, as well as the opposite effect of ITIM and ITAM, are typical equilibrium mechanisms that determine the direction in which microglia respond to environmental changes and maintain the functional balance of microglia.

### 2.1 Triggering receptor expressed on myeloid cells (TREM)

TREM is composed of a group of cell surface innate immune receptors of the immunoglobulin superfamily that are expressed on various myeloid cell populations throughout the body (microglia in the brain), which play an important role in regulating myeloid inflammatory activity. There are seven human TREM family members, namely TREM1‐3 and TREML1‐4, and the most studied are TREM1 and TREM2.

TREM1 is mainly expressed in mononuclear macrophages in peripheral nervous system and microglia in the central nervous system.^63^ In recent years, studies have found that TREM1 is poorly expressed in the resting state. However, under the action of injury stimulation, it can be expressed upregulated and synergistically amplify the inflammatory response mediated by multiple PRR, resulting in uncontrolled inflammation. Microglia are also highly expressed in TREM1 upon activation,^64^, ^65^ which can mediate neuroinflammation in ischemic stroke by interacting with the spleen tyrosine kinase (SYK) signaling pathway, activating downstream NF‐κB and nucleotide‐binding oligomerization domain (NLRP3) inflammasomes. Activation of NLRP3 inflammasome by TREM1 also plays a key role in neuroinflammation following subarachnoid hemorrhage (SAH),^66^ and inhibition of TREM1 reduces neuroinflammation and improves neurological deficits.^67^ It can also improve ischemic stroke‐induced neuronal damage and reduce microglia‐mediated neuroinflammation by reducing oxidative stress and thermochemistry.^68^ TREM1 also plays a significant role in the inflammatory response of neurodegenerative diseases.^69^

The above studies suggest that TREM1 plays a very important role in the occurrence and development of central nervous system diseases by enhancing the immune inflammatory response process of microglia, regulating the inflammatory microenvironment of diseases, inflammatory cell infiltration, cytokine production, blood‐brain barrier destruction, and strengthening the phagocytic ability of phagocytes. However, there are few studies on TREM1 in PND. At present, only one literature has been searched from domestic and foreign databases to introduce the upregulation of TREM1 expression in peripheral blood after knee arthroplasty in elderly patients, and TREM1 mediates the occurrence of PND by regulating the inflammatory response.^70^ However, this study restricts itself to the phenomenon level and does not involve mechanism research. Therefore, it is necessary to further explore the function and mechanism of TREM1 in PND.

TREM2 is highly expressed in myeloid cells, such as dendritic cells, macrophages, neutrophils, and microglia, and plays a role in regulating cell differentiation, phagocytosis, and chemotaxis. The study found that TREM2 may reduce anesthesia and surgically induced spatial learning and memory impairment in aged C57/BL6J mice by activating mitophagy and inhibiting NLRP3 inflammasomes.^22^ TREM2 can also alleviate microglia‐mediated neuroinflammation by modulating PI3K/AKT signaling, thereby alleviating PND.^23^ The selective agonist of TREM2, Heat Shock Proteins 60 (HSP60), can induce the upregulation of TREM2 expression in the mouse brain, alleviate neuroinflammation, reduce apoptosis of mouse neuronal cells, and improve mouse learning and memory. Eliminating the increase of TREM2 expression induced by HSP60 with TREM2 small interfering RNA (siRNA) can block the protective effect of HSP60.^24^

### 2.2 Cluster of differentiation 200 receptor (CD200R)

CD200R is also known as the OX2 receptor, and together with the ligand CD200 (OX2), it is a cell surface glycoprotein containing two immunoglobulin domains. CD200R is mainly expressed in microglia, and its ligand CD200 is a membrane protein expressed in neurons, which together participate in the communication between microglia and neurons, with powerful immunomodulatory functions. The CD200‐CD200R interaction helps maintain the quiescent state of microglia, and CD200 deficiency can lead to microglia activation and polarization to M1 type.^25^ Exercise significantly increases CD200 and CD200R levels in the ipsilateral hippocampus and cortex after stroke. Exercise improves the inflammatory environment after stroke by activating the CD200/CD200R signaling pathway, which has a beneficial effect in promoting neurogenesis and functional recovery.^26^ In the PND model, hippocampal CD200 messenger RNA levels in adult and elderly rats decreased on postoperative Day 1 and improved on postoperative day.^27^ CD200 fusion protein (CD200‐Fc) can activate CD200R, improve age‐related M1 microglia activation, and improve synaptic function.^28^ At present, the role of CD200R activation in promoting microglia M2 type has become a focus. Stimulation of activated microglia with CD200R agonists produces anti‐inflammatory cytokines (IL‐10, IL‐4, etc.), while inhibition of CD200R prevents microglia from transitioning to an anti‐inflammatory phenotype.^29^ The study found that the reduction of CD200 expression in neurons of aging rats was associated with synaptic dysfunction and long‐term potentiation (LTP) and could be reversed by giving CD200‐Fc.^30^ Activation of CD200R attenuated neuroinflammatory response and cognitive decline in mouse models of PND, increasing the expression of PSD‐95.^31^ The CD200‐CD200R axis plays an important role in synaptic function in neurons.

## 3 PRR 

PRRs are known to mediate microglial activation and recognize molecular motifs consisting of pathogen‐associated molecular patterns (PAMPs) and damage‐associated molecular patterns (DAMPs). PAMPs consist of a group of molecular determinants of invading pathogenic bacteria, fungi, or viruses. DAMPs are derived from intracellular proteins/enzymes, cellular debris, and so forth released from necrotic tissue after diseases, such as ischemic injury and neurological disorders. Studies have found that toll‐like receptors (TLRs), receptor for advanced glycation end products (RAGE), carbohydrate‐binding receptors (CBR), and scavenger receptors (SRs) in the PRR family are all associated with central nervous system damage. The relationship between TLRs and PND has been described in detail in several previous studies and will not be discussed here.^32^, ^33^, ^71^

### 3.1 RAGE

RAGE is named for its binding to advanced glycation end products, a pattern‐recognition receptor that is highly expressed in immune cells. In many studies, RAGE has been considered an amplifier of the immune inflammatory response. It regulates the expression of proinflammatory factors by activating the MAPK and NF‐κB signaling pathways, which play an important role in inflammation, oxidative stress, and cell dysfunction.^34^ Zhang et al. found that inhibition of the HMGB 1/RAGE signaling pathway reduced microglia‐mediated neuroinflammatory response.^35^ He et al. have shown that anesthesia and surgical trauma can upregulate the expression of proinflammatory cytokines (HMGB 1, TNF‐α, and RAGE). HMGB1 and RAGE signaling pathways contribute to the change of blood‐brain barrier permeability after peripheral surgery trauma, which may be a key mediator of cognitive decline induced by anesthesia surgery, leading to neuronal damage and cognitive dysfunction in elderly rats.^36^ RAGE plays an essential role in the inflammatory process of PND, and blocking RAGE can inhibit neuroinflammation and weaken PND.^37^ Wang et al. found that Dexmedetomidine may reduce the surgical‐induced hippocampal inflammatory response and reduce the occurrence of PND by regulating M1/M2 microglial polarization and inhibiting the HMGB 1/RAGE/NF‐κB signaling pathway.^72^

### 3.2 CBR

Recent studies have shown that the carbohydrate structure formed during posttranslational modification of proteins can interact with microglia to regulate the activation state and function of microglia. Understanding the relationship between carbohydrate molecules and CBRs on microglia may provide new ways to mitigate inflammation‐induced damage in various neurological diseases. Galectin 1 (Gal‐1) and galectin 3 (Gal‐3) on microglia regulate the inflammatory response. The former promotes the anti‐inflammatory response, and the latter promotes the proinflammatory cascade.^38^ As a β‐galactoside‐binding protein, Gal‐1 has a variety of biological activities and is a key regulator of neuroinflammation. Gal‐1 reduces the secretion of proinflammatory cytokines (IL‐1β, TNF‐α, iNOS, etc.) through the MAPK/IκB/NF‐κB signaling pathway, inhibits microglial activation in PD models, and improves neurodegenerative processes.^39^ In vitro, Gal‐1 regulates the function of microglia by reducing LPS‐stimulated proinflammatory cytokines (NO, TNF‐α, etc.) and helps increase the secretion of anti‐inflammatory cytokines (TGF‐β, IL‐10, etc.). It improves autoimmune inflammation and inactivates M1 microglia, suggesting that Gal‐1 may delay neurodegeneration.^73^ Gal‐1 inhibits LPS‐induced inflammation of BV2 microglia by inhibiting the expression of IRAK1 and reducing the translocation of NF‐κB p65 and c‐Jun. In addition, Gal‐1 can reduce the expression of proinflammatory cytokines (IL‐1β, IL‐6, and TNF‐α), inhibit the inflammatory activation of microglia, and prevent hippocampal neuronal damage, thereby reducing PND in elderly mice. Gal‐1 may have a protective effect against surgery‐induced neuroinflammation and neurocognitive impairment.^74^ Microglia Gal‐3 expression is significantly increased in the early stages of spinal cord injury.^75^ Patients with traumatic brain injury (TBI) also had an increased release of Gal‐3 from cerebrospinal fluid and plasma,^76^, ^77^ and there was a positive correlation between Gal‐3 levels in plasma and Glasgow Coma Scale scores, suggesting that Gal‐3 may be a potential biomarker for TBI. In the cerebral hemorrhage model, Gal‐3 levels increase in the cerebral hemorrhage region from Days 3 to 7 after injury, and Gal‐3 is expressed mainly in proinflammatory microglia.^78^ Gal‐3 has been found to be necessary for microglia activation and proliferation after ischemic injury, and gene deletion of Gal‐3 leads to reduced cytokine release 8 days after ischemia and prevents neurodegeneration.^79^

### 3.3 SRs

So far, six families of SRs have been identified, from SR‐A to SR‐F. The roles of SR‐A, SR‐BI, CD36, CD68, and SR MARCO in AD have been described to varying degrees in previous studies, of which CD36 has been the most studied. As a member of the SR‐B family, CD36 is mainly expressed in macrophages and microglia and is involved in apoptotic cell uptake, signal transduction, cell adhesion, angiogenesis, and immune function.^80^ CD36 regulates the uptake of myelin debris by macrophages and microglia through the nuclear factor erythroid2‐related factor 2 (Nrf2) signaling pathway, inhibits CD36, and reduces the uptake of myelin debris, which can promote neuroinflammation in vitro and in vivo.^81^ CD36 has been identified as an essential molecule in amyloid β‐protein (Aβ) recognition microglial activation.^82^ Inhibition of CD36 interaction with Aβ causes a decrease in proinflammatory cytokine production and reactive oxygen species (ROS) production.^83^ TREM2 induces CD36 upregulation, enhances the phagocytosis of Aβ, and protects neuronal cells from Aβ‐induced cytotoxicity.^84^ In the late stage of AD, CD36 promotes neurodegeneration by stimulating the production of ROS and increasing the deposition of Aβ. The increased Aβ also stimulates CD36 expression, causing excessive ROS production that directly kills cells, especially fragile neurons.^40^ Previous studies have shown numerous similarities in the pathogenesis of PND and AD. Therefore, SRs may be the future research direction of PND.

## 4 PURINERGIC RECEPTORS

Purinergic receptors are divided into two main classes based on their binding properties, P1 and P2. P1 receptors bind adenosine, whereas P2 receptors bind ATP.^85^

### 4.1 P1 receptors

The P1 receptor is a class of G protein‐coupled seven transmembrane proteins. There are four subtypes: A1, A2A, A2B, and A3, which perform different or overlapping functions. Although extracellular adenosine concentration is shallow under physiological conditions, it plays an important role in regulating brain functions, including sleep, arousal, movement, anxiety, cognition, and memory. Under pathological conditions, such as hypoxia, ischemia, or trauma, adenosine can be released from injured neurons and other types of cells. All four isoforms of adenosine receptors are expressed on microglia and regulate microglial activity, such as proliferation, apoptosis, protein production, and chemotaxis.^41^

Cordycepin can upregulate the level of A1R in rats with cerebral ischemia, reduce dendritic morphological damage in the CA1 region of the hippocampus, improve herniated transmission, and improve learning and memory ability.^42^ Mice administered with A1R agonists or antagonists and conventional A1R knockout mice were exposed to chronic intermittent hypoxia to determine the function of A1R signaling. it was found that morphological damage and apoptosis of hippocampal neurons induced by chronic intermittent hypoxia were aggravated by A1R antagonists or A1R gene deletion, and A1R agonists partially alleviated learning and memory function. Activation of A1R signaling can protect hippocampal neurons and cognitive function from chronic hypoxia damage.^43^

A2AR promotes the phosphorylation of CREB via the PKA pathway and inhibits the production of proinflammatory cytokines.^44^ A2A receptor inhibitor can improve the cognitive function of Parkinson's patients.^45^ Studies have shown that A2AR activation is essential for the function of neurotrophic receptors at synapses.^46^ Activating A2AR increases the expression of vascular endothelial growth factor and brain‐derived neurotrophic factor after lipopolysaccharide (LPS) and sevoflurane exposure improves damaged cells, and ultimately increases cell viability in the PND environment.^47^

The roles of A2B and A3R in PND are currently unclear.

### 4.2 P2 receptors

There are five subtypes of P2 receptors: P2X, P2Y, P2Z, P2U, and P2T. Microglia express P2X and P2Y. P2X7R is an ATP‐gated transmembrane ion channel receptor. A large amount of ATP can be released during cell damage, and long‐term, high concentrations of ATP can activate P2X7R and induce the release of inflammatory factors.^48^ Activation of microglial NLRP3 inflammasome depends on activation of P2X7R. After P2X7R was blocked, the expression of NLRP3 and p20 was significantly inhibited. Surgery‐induced neuroinflammation and cognitive decline can be reversed by applying P2X7R antagonists or P2X7R knockout mice.^5^ Upregulation of P2X4 receptors has been found to activate microglia, produce multiple proinflammatory cytokines, and promote neuroinflammation, ultimately inducing neurotoxicity and neuronal cell damage.^49^, ^50^ P2X4 receptors are involved in various neuroinflammatory diseases, such as ischemic stroke^51^ and neuropathic pain.^52^ In the ischemic stroke model, intraperitoneal injection of the P2X4 inhibitor 5‐BDBD significantly reduced the activation of microglia and the expression of P2X4 in the cerebral infarction area, achieving neuroprotection.^53^ 5‐BDBD reduces postoperative hippocampal P2X4 expression and microglia activation, reduces inflammation, and reverses cognitive impairment. Postoperative neuroinflammation and cognitive impairment are associated with activation of the P2X4/NLRP3 signaling pathway in the hippocampus, and inhibition of this pathway may be a way to prevent and treat PND in the future.^54^

The function of P2Y in PND is currently unclear.

## 5 CHEMOKINE RECEPTORS

Chemokine receptors are a class of G protein‐coupled receptors. When chemokines and chemokine receptors interact, they control multiple functions and the migration of immune cells. Currently, the most common chemokine receptors associated with microglial function in central nervous system injury have been found to be CX3CR1, CXCR3, CXCR4, CCR2, and CCR5, of which CX3CR1 and CCR2 are the most studied in PND.

### 5.1 CX3CR1

Studies have found that CX3CR1 regulates various microglial functions, including adhesion, migration, proliferation, production, and clearance of inflammatory cytokines.^86^ Under physiological conditions, disruption of CX3CR1 signaling through increased action of IL‐1β leads to impaired cognitive function and synaptic plasticity,^87^ suggesting that CX3CR1 plays an important role in maintaining central nervous system homeostasis and normal interneuronal information transmission. Inhibiting CX3CR1 with siRNA exacerbates ischemia‐induced microglial activation, enhances IL‐1β expression, and worsens ischemia‐induced cognitive impairment.^55^ However, there is no significant effect on ischemia‐induced hippocampal neurodegeneration, possibly due to regional heterogeneity of microglia resulting in different sensitivity to the same pathological signal.^56^ Recent studies have found that Tau can bind directly to CX3CR1 and then compete with the receptor's native ligand, leading to disruption of neuron‐glial signaling that decouples microglial activation.^57^ Inhibition of CX3CR1 signaling reduces mechanical pain and increases proinflammatory cytokines levels, improving PND.^58^

### 5.2 CCR2

The primary function of CCR2 is to guide microglia to migrate to the injury site. CCR2 and/or its ligands are upregulated in many central nervous system injury types, including ischemia, hemorrhage, trauma, and hypoxia. The surgery increases the level of hippocampal proinflammatory cytokines and induces CCR2 cells in the peripheral circulation into the hippocampus, where microglia are activated and participate in the process. Perioperative microglial depletion eliminates the surgical‐induced increase in hippocampal proinflammatory cytokines and the influx of CCR2 cells into the hippocampus.^17^ Tibial fracture surgery increases microglial activation and upregulates the expression of CCR2, inducing learning and memory impairment. Pretreatment with the CCR2 antagonist RS504393 improves cognitive function by reducing microglial activation and polarization to the M1 type, inhibiting neuronal damage and inflammatory cytokine production.^59^

In addition, it was found that JWH133, an agonist of cannabinoid receptor CB2, can improve the learning and memory of PND mice by promoting the activation of microglia in hippocampus and prefrontal cortex and the secretion of anti‐inflammatory cytokines.^60^ Proteomic analysis of cerebrospinal fluid in patients with PND revealed that 17 differential proteins were associated with complement receptors, suggesting that complement receptors may play an important role in PND.^61^ Histamine binding to its receptors H2 and H3 can inhibit microglial overactivation and proinflammatory factor expression through the PI3K/AKT/FoxO1 pathway, alleviating neurocognitive damage after laparotomy in elderly rats.^88^

## 6 CONCLUSION

**Figure 2:**
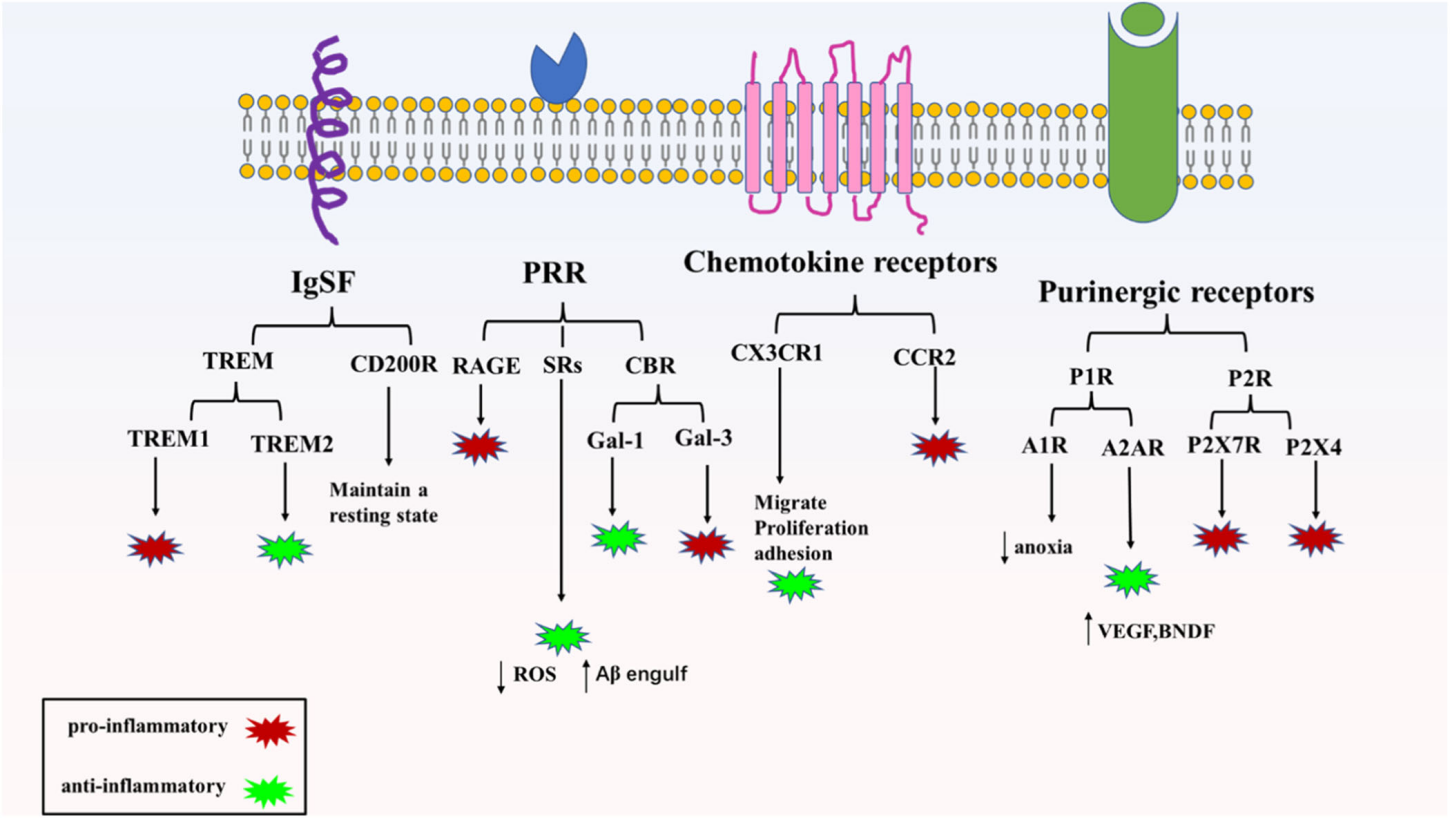
Microglial surface receptors were reported to be involved in PND. CBR, carbohydrate‐binding receptors; CD200R, cluster of differentiation 200 receptor; IgSF, immunoglobulin superfamily; PND, perioperative neurocognitive disorders; PRR, pattern recognition receptors; RAGE, receptor for advanced glycation endproducts; SRs, scavenger receptors; TREM, triggering receptor expressed on myeloid cells. [Color figure can be viewed at wileyonlinelibrary.com]

Microglial overactivation‐mediated neuroinflammation plays an important role in the development of PND. Microglia have many types of surface receptors and complex functions, which are important media for microglia to receive external stimulation and intercellular signal transmission and realize the various functions of microglia through coordination. Among them, immunoglobulin superfamily receptors, chemokine receptors, purinergic receptors, PRRs, and so forth are all involved in the occurrence and development of PND to varying degrees (Figure 2). After different receptors on the surface of microglia receive external stimuli, microglia are polarized into different phenotypes. It can promote and inhibit the occurrence of PND by promoting the inflammatory reaction and inhibiting the inflammatory reaction, but the specific mechanism is still unclear. The study of microglial surface receptors is of great significance for understanding the pathogenesis of PND and discovering potential biomarkers and new therapeutic targets. The above receptors may be targets for mitigating PND and will become the focus of future research.

## AUTHOR CONTRIBUTIONS

Chun‐Chun Tang collected material and composed the paper, and Zhao‐Qiong Zhu and De‐Xing Liu modified the review.

## CONFLICT OF INTEREST STATEMENT

The authors declare no conflict of interest.

## ETHICS STATEMENT

The authors have nothing to report.

## Data Availability

Data sharing is not applicable to this article as no data sets were generated or analyzed during the current study.
